# Assessing Health Care Providers’ Knowledge and Practices of Nutrition during Pregnancy in Lebanon: A Cross-Sectional Study

**DOI:** 10.3390/medicina59081471

**Published:** 2023-08-16

**Authors:** Jessy Rizk, Eleni Andreou, Dona Hileti, Ali Ghaddar, Antonis Zampelas

**Affiliations:** 1 Department of Life Sciences, University of Nicosia, Nicosia CY-2417, Cyprus; rizk.j@live.unic.ac.cy (J.R.); andreou.el@unic.ac.cy (E.A.); hileti.d@unic.ac.cy (D.H.); 2 Department of Biomedical Science, Lebanese International University, Beirut 146404, Lebanon; ali.ghaddar@liu.edu.lb; 3 Public Health Research Group, University of Alicante, 03690 Alicante, Spain; 4 Laboratory of Dietetics and Quality of Life, Department of Food Science and Human Nutrition, Agricultural University of Athens, 11855 Athens, Greece

**Keywords:** nutrition, pregnancy, health care providers, knowledge

## Abstract

*Background and objectives:* Health care professionals (HCPs) are well-positioned to discuss healthy behaviors during pregnancy, but the published research of prenatal healthcare providers’ knowledge about the significance of nutrition during pregnancy in Lebanon is scarce. The purpose of this study was to explore the knowledge, attitudes, and practices of Lebanese prenatal healthcare providers towards nutrition during pregnancy. *Materials and Methods:* A cross-sectional study using an online questionnaire was conducted. Health care providers were contacted by phone and email to participate in the online survey. A list of all clinics providing antenatal health services was obtained from the Order of Physicians and the Order of Midwives. A multistage random sample was selected. In the first stage, it was stratified per region (Beirut center or suburbs, and the southern region). In the second phase, it was stratified per clinic type (private, primary healthcare center, or hospital). Gynecologists and midwives who are members of the Order of Physicians and the Order of Midwives (n = 1333), were included. *Results:* Two-hundred and six responses (55% males) were obtained. Approximately 44% of the HCP were aged 50 and older, and 68.4% had more than 10 years of work experience. HCPs from Beirut represented 41.3% of the respondents. Eighty-eight percent of the HCPs were physicians, and 11% were midwives. The majority of the participants considered nutrition during pregnancy to be very important. Furthermore, half of these participants considered their position in delivering nutrition information as very significant. Most of the respondents reported that they provide nutrition advice to pregnant women, and they also received nutrition education during their profession. However, they perceived their nutrition knowledge as inadequate. *Conclusion:* Health care providers’ attitude towards the importance of maternal nutrition and their confidence in talking about nutrition-related topics with pregnant women were positive despite the lack of knowledge in several areas related to nutrition during pregnancy. Therefore, there is a need for continuing nutrition education for health care providers and the implementation of nutrition education programs to achieve better health outcomes.

## 1. Introduction

Appropriate nutrition is needed, not only for the growth and development of the fetus and the health of the mother during pregnancy, but also for the long-term health effects of both the mother and the child at later stages of their lives [1]. The prenatal period is a time when several metabolic systems can be influenced by conditions of maternal physiology and environmental exposures, including dietary nutrient intake [2]. Moreover, nutrition behaviors during pregnancy, high pre-pregnancy body weight, and maternal iron deficiency can impact the health of the mother and offspring [3]. To reduce these outcomes, weight and dietary counseling during pregnancy and postpartum should be prioritized [4].

Despite the evidence of the benefits of a healthy lifestyle during pregnancy, women’s actual behaviors differ [5]. Some explanations for this inconsistency could be the limited nutrition information provided by health care professionals (HCPs), the inadequate counseling, and also the fact that the guidelines could be overly broad and/or ineffectively disseminated through counseling practices [5]. There is also evidence that although the majority HCPs in antenatal clinics have adequate awareness of nutrition during pregnancy, nutrition education services are considered inadequate [6]. In addition, midwives also lack knowledge of basic nutrition guidelines for pregnant women. This might be due to the inadequate nutrition education provided in midwifery programs [7]. Likewise, it has been reported that medical students lack basic nutrition courses during their education to provide effective nutrition care to patients [8].

In the Middle East, a limited number of studies examined the knowledge and practices of women regarding periconceptional folic acid supplementation [9,10,11]. One assessed the adequacy of nutrient intakes among pregnant women compared to the Dietary Reference Intakes (DRIs) [12], while others correlated maternal nutrition and lifestyle characteristics with birth outcomes [13,14].

In Lebanon, only one study assessed the knowledge, attitudes, and practices of primary care physicians regarding nutrition counseling, and it also observed barriers to nutrition counseling, such as gaps in physician’s nutritional knowledge [15]. However, there are no studies investigating health care providers’ nutrition knowledge and practices specifically during pregnancy.

Therefore, the aim of the present study was to investigate the nutrition knowledge of HCPs practicing in Lebanon, their attitudes towards the significance of nutrition during pregnancy, and their confidence in communicating to pregnant women the nutrition related issues that can occur during gestation.

## 2. Materials and Methods

### 2.1. Study Population

The study involved prenatal healthcare providers, including physicians and midwives, practicing in Lebanon. 

The sample was selected from Beirut and the southern region, which are two out of the eight governorates in Lebanon. A list of all clinics providing antenatal health services was obtained from the Order of Physicians and the Order of Midwives. A multistage random sample was selected. In the first stage, it was stratified per region (Beirut center or suburbs, and southern region). In the second phase, it was stratified per clinic type (private, primary healthcare center, or hospital). This stratification was conducted in order to ensure a heterogeneous group of clinics with different socio-economic status.

Two-hundred clinics, which represent 40% of the registered clinics in the Lebanese Society of Obstetrics and Gynecology and the Lebanese Order of Midwives, were selected, and the sample size of HCPs required was estimated to be a minimum of 200 (from a total population of 500) to ensure sufficient statistical power using a margin error of 5 and a confidence level of 95%.

### 2.2. Questionnaire

The questionnaire adopted for this study was based on previous literature where researchers developed it to learn more about the nutrition-related conditions and knowledge of pregnant women during gestation [16,17,18]. In order to verify the consistency of the questionnaire, a small pilot study was conducted among 30 individuals, and modifications were applied accordingly. The alpha–Cronbach’s coefficients were calculated to assess the reliability of the questionnaire.

The questionnaire used to explore prenatal healthcare providers’ nutrition knowledge during pregnancy was based on an existing survey that explored Australian midwives’ nutrition knowledge [19] and included different domains: the nutrition education section, the attitudes and level-of-confidence section, the practices section, and the demographics section. In the nutrition education section, HCPs were asked to report if they had attained nutrition education during their profession, and if so, how they received it and what was the content covered. The questions asked in the attitudes section concerned HCPs’ involvement in providing nutrition information, their rating for the significance of nutrition, and their confidence in talking about nutrition-related problems with pregnant women. In the practices section, HCPs were asked about their perception regarding the best timing to start talking about nutrition-related problems to pregnant women, their sources of nutrition information, and if they refer their patients to dietitians. In the pregnancy knowledge section, HCPs were asked 12 questions (either single answer or multiple answers) to determine their knowledge of energy requirements during the trimesters of gestation, the appropriate gestational weight-gain range, the vitamin to be supplemented in case of a vegetarian mother, the amount and timing of folic acid supplementation, the calcium and iron requirements, the sources of dietary iron, how to avoid listeria poisoning, how to reduce nausea and vomiting, and how to resolve constipation during pregnancy. The last section of the survey covered demographic data (education, age, sex, place of practice, years of experience, etc.) of the respondents.

### 2.3. Data Analysis

Data were analyzed using SPSS (Version 22.0). Descriptive and inferential statistics were used to describe and analyze the data, respectively. One-way ANOVA and independent *t*-test, *p* < 0.05, were applied to evaluate the differences in the mean overall scores of nutrition knowledge based on categories of demographic factors. Three linear regression analysis models were used to observe the effects of independent variables on knowledge as dependent variables (Model 1), attitudes (model 2), and practices (Model 3). The factors that were included in the regression analysis are the ones that were significantly associated with participants’ nutrition knowledge (in one-way ANOVA and independent *t*-test, *p* < 0.05).

### 2.4. Ethical Approval

The study obtained ethical approval from the IRB of the Researcher’s University (Lebanese International University). Informed consent of participants was obtained. Participation was voluntary. Confidentiality was assured, and no names were used in the survey.

## 3. Results

### 3.1. Demographic Characteristics

The demographic characteristics of the respondents are shown in Table 1. Two hundred and six HCPs participated in the study. Approximately 44% of respondents were females and 55% were males. Approximately 44% of the HCP were aged 50 and older, and 68.4% had work experience of more than 10 years. HCPs from Beirut represented 41.3% of the respondents. Out of the HCPs who filled out the survey, 88% were physicians and 11% were midwives. The majority of the HCPs (~70%) had a dietitian’s service or support for pregnant women at their clinic or hospital.

### 3.2. General Nutrition Knowledge for Pregancy

Table 2 shows the answers to the nutrition knowledge questions. Most HCPs correctly identified the appropriate timing and amount of folic acid supplementation (85% and 72.8%), as well as the energy requirement changes during the trimesters of gestation (84.5%). Half of the HCPs answered correctly the questions related to the energy and iodine requirements during pregnancy, and the vitamin supplement needed in case pregnant women were vegetarian (57.8%, 52.9%, and 50.5%, respectively). The lowest scores were for the questions that assessed their knowledge of the healthy weight-gain range during pregnancy and the daily recommended servings of dairy foods (75.2% incorrect responses for both questions).

The majority of the respondents (82.0%) were knowledgeable about the foods that would help resolve constipation during pregnancy. Around one-third of the respondents (35.9%) were able to identify the appropriate advice to help manage nausea and vomiting that could occur during pregnancy. The questions with the lowest percentage of correct answers firstly concerned the types of food to avoid to lower the risk of listeria contamination and, secondly, the dietary sources of iron (14.6% and 9.2%, respectively).

### 3.3. Nutrition Education

The majority of the HCPs reported that they had obtained nutrition education during their profession; 16% answered “No”, while 3.9% did not remember receiving this education. Around two-thirds of the HCPs stated they obtained nutrition education related to general nutrition information, weight management during pregnancy, and nutrition-related issues such as constipation. The majority of the HCPs (78.2%) considered that receiving more nutrition information would benefit their practice.

### 3.4. Attitude and Confidence

The majority of HCPs considered nutrition to be very important during pregnancy, but only half classified their role to be very significant in providing nutrition information to pregnant women (Table 3).

Around 60% of HCPs were very confident in providing pregnant women the following: general nutrition advice, weight-gain recommendations, and, lastly, advice on vitamins, obesity, and diabetes mellitus. Additionally, HCPs had a moderate level of confidence in talking about vegetarian diets, 32.5% of them were very confident in providing dietary advice to women with medical conditions, and 43% of them were very confident in discussing post-natal nutrition (breastfeeding).

When HCPs were asked about the weight category, 90% preferred their patients to have normal body weight (body mass index (BMI) of 18.5–24.9 kg/m^2^), while only 8.7% reported that they do not mind the body weight. The main reason behind the preference of a normal body weight was because HCPs believe that women who are not at a normal body weight might experience complications during pregnancy (72.8%).

### 3.5. Practices

The practices of HCPs towards providing nutrition advice, talking about nutrition problems with pregnant women, and their origin of information for their advice are shown in Table 4. The majority of the respondents were providing nutrition advice to pregnant women. The occasions when HCPs were talking about nutrition problems with pregnant women were when pregnant women had a medical condition that required nutrition intervention. One-third of HCPs would talk about nutrition problems with pregnant women at every antenatal visit. However, 13.1% of HCPs would discuss nutrition issues at the first antenatal visit, and only 7.8% when the pregnant woman asked questions.

The two most-used information sources by the HCPs as the basis of their nutrition advice were dietitians (32.0%), followed by general knowledge and medical/midwifery education (30.1%).

The majority of HCPs (72.3%) referred pregnant women to a dietitian.

Table 5 shows the bivariate analysis for the total score per the study variables. The mean of the total score was 11.7 out of 20. The only significant difference was by the age group, with higher scores achieved for those between 41–50 years of age.

In the multiple regression analysis, shown in Table 6, the independent variables were entered simultaneously into the model to investigate their effect on total knowledge scores (Model 1), providing nutrition advice (Model 2), and discussing nutrition issues (Model 3). In Model 1, midwives had significant lower total knowledge scores compared to physicians (*p* = 0.036). As for Model 2, the higher the total knowledge scores, the higher possibility of HCPs to provide nutrition advice (*p* = 0.003). Regarding Model 3, the higher the total knowledge scores, the lower the HCPs who discussed nutrition issues (*p* = 0.009). No significant differences were observed for the other independent variables (age, gender, years of experience, and dietitian’s service).

## 4. Discussion

The present study explored four main areas related to prenatal nutrition education by HCPs in Lebanon, namely nutrition education, attitudes, practices, and knowledge. Most of the HCPs in this study viewed nutrition during pregnancy to be very important, while half of the HCPs considered their part in communicating nutrition information to pregnant women to be very significant. In addition, although the majority of HCPs were providing nutrition advice to pregnant women, their nutrition knowledge was inadequate in the following areas: in the healthy weight-gain range during pregnancy, the daily recommended number of servings of dairy foods, the foods that should be avoided as a risk for listeria in pregnancy, the advice to reduce the effect of nausea and vomiting during pregnancy, and the food sources of iron. The majority of HCPs reported receiving nutrition education during their career/practice, but only half of the HCPs were very confident in providing general nutrition advice to pregnant women.

Similar to the results of our study, Stang et al. also reported that although HCPs perceive nutrition education to be important [20], their nutrition knowledge was inadequate in several areas. In other studies, it was also observed that physicians are generally aware of nutritional topics but have poor knowledge in important areas of nutrition, including nutrition guidelines during pregnancy [21,22,23]. Consequently, the lack of nutrition knowledge and of the time to be dedicated during antenatal care services are considered barriers for the provision of proper nutritional advise [24]. In our study, the main factor for the efficiency in nutrition advice was age (41–50 years). This was because HCPs in this particular age group had higher scores of nutrition knowledge compared to the other HCP age groups.

HCPs would talk about nutrition problems with pregnant women mainly if the pregnant woman had a medical condition, consequently requiring nutrition counseling, and only a third of HCPs would talk about nutrition problems at every antenatal visit. A possibility for this finding could be that guidelines are not being effectively disseminated through HCP counseling practices [5].

The level of knowledge of HCPs regarding the average change in energy requirements during pregnancy was low. The Institute of Medicine states that extra energy is needed during pregnancy for the fetal, placental, and maternal tissue growth [25], but for the calculation of the total energy needs, the potential declines in physical activity has to be considered [26].

HCPs’ knowledge of appropriate weight gain during pregnancy was low, which is of concern knowing the association between excessive gestational weight gain and the higher risks of maternal-, fetal-, and childhood-adverse outcomes [27]. It is noteworthy that the American College of Obstetricians and Gynecologists recommend calculating BMI at the first antenatal visit in order to provide effective diet and exercise counseling [28]. Physicians who are unaware of the recommendations are unlikely to advise their patients about weight gain during pregnancy [29]. This is possibly one of the main reasons why the majority of pregnant women report either that they are not informed, or that they are misinformed about GMG recommendations [30,31]. Barriers for an effective counseling on weight gain during pregnancy are reported to be the weak HCP knowledge of the guidelines [32], the low priority of counseling on appropriate weight gain, the lack of resources, and the belief that any advice provided to pregnant women is unlikely to be followed [33].

Gestational weight gain is not the only issue of concern regarding body weight. Pre-pregnancy obesity is also associated with a higher risk of complications during pregnancy (gestational diabetes, macrosomia), at delivery (caesarean section, shoulder dystocia), and postpartum (morbidity and mortality). Even though these findings are well-reported, it has also been observed that 46.5% of women gained excessive body weight during pregnancy, and that women with high pre-pregnancy BMI were more likely to gain excessive weight than women who were of normal weight [34]. Most HCPs do not usually address the adverse effects linked to abnormal GWG [35].

In Lebanon, HCPs widely use, as a reference, the IOM guidelines [36,37,38]. In the first study, an excessive GWG among the overweight and obese women was observed, with almost half of them exceeding the guidelines set by IOM [36]. In the second study, an increase of 1.36 kg/m^2^ in the BMI of women in child-bearing age was reported [37], and in the third study, 42% of women were entering pregnancy with a BMI exceeding 25 kg/m^2^ [38].

The knowledge of HCPs regarding nutrient requirements during pregnancy varied. As for the timing and amount of folic acid supplementation needed, the knowledge was adequate. That could be due to the WHO recommendation that all women trying to conceive should take a folic acid supplement until 12 weeks of gestation [39]. In addition, the knowledge about the requirements for Vitamin B_12_ and iodine intakes was low, in agreement with the study of McMullan et al. [40].

Finally, when the various factors were combined in multiple regression (i.e., total knowledge score, providing nutrition advice, and discussing nutrition issues), the factor that was significant was that physicians possessed better nutrition knowledge than midwives. Moreover, HCPs with a higher total score were providing nutrition advice but were discussing nutrition issues less with their patients. It is of paramount importance that HCPs should refer pregnant women to a dietitian for detailed and professional nutritional counseling.

The study has some limitations. First, the cross-sectional nature of the study makes generalization of results hindered, and so results should be inferred with care. HCPs who agreed to fill out the survey might have been more interested or knowledgeable about nutrition than HCPs who chose not to participate. The number of physicians who participated in this study was much higher than the number of midwives. Finally, the study relied on a questionnaire that was self-administered and self-reported, thus the findings could be influenced by response bias such as over-reporting nutrition counseling practices (providing and discussing nutrition issues with pregnant women).

## 5. Conclusions

Nutrition during pregnancy is important as it may affect the health of the mother and baby not only during pregnancy but also later in life. Prenatal HCPs play a vital role in providing nutrition advice to pregnant women. Continued nutrition education that provides evidence-based nutrition guidelines would support HCPs in practice. HCPs should also refer the pregnant women to a dietitian for detailed dietary advice. Moreover, developing nutrition education programs by community dietitians that target women and HCPs could improve nutrition knowledge and practices to achieve better health outcomes. Policymakers should better position nutrition as a matter of importance during pregnancy by incorporating nutrition counseling during prenatal visits.

## Figures and Tables

**Table 1:** Demographic characteristics of the study participants.

	Health Care Professionals (n = 206)
**Age, years**	
21–30	9 (4.4%)
31–40	60 (29.1%)
41–50	44 (21.4%)
>50	91 (44.2%)
**Sex**	
Female	90 (43.7%)
Male	114 (55.3%)
**Profession**	
Physician	183 (88.8%)
Midwife	12 (5.8%)
Hospital based training midwifery	2 (1.0%)
Initial midwifery degree	5 (2.4%)
**Experience, years**	**n = 205**
<2	4 (1.9%)
2–5	21 (10.2%)
6–10	39 (18.9%)
>10	141 (68.4%)
**Territory of work**	
North	7 (3.4%)
Mount Lebanon	61 (29.6%)
Beirut	85 (41.3%)
Bekaa	15 (7.3%)
South	29 (14.1%)
Nabatieh	7 (3.4%)
**At your service, do you have a dietitian’s support for pregnant women?**	
Yes	144 (69.9%)
No	62 (30.1%)

**Table 2:** Percentage of participants who answered correctly and incorrectly to the nutrition knowledge questions.

	Correct Answer	Incorrect Answer
**One-answer questions related to knowledge**	n (%)	n (%)
**Change in caloric needs during pregnancy**	119 (57.8)	82 (39.8)
Do the caloric needs of pregnant women differ during the trimesters of pregnancy?	174 (84.5)	32 (15.5)
The range of healthy weight gain during pregnancy for a woman who started her pregnancy at normal weight	50 (24.3)	155 (75.2)
The most important vitamin supplement for vegetarian pregnant women	104 (50.5)	102 (49.5)
When should women take folic acid supplement?	175 (85.0)	31 (15.0)
The amount of folic acid supplements needed daily during pregnancy	150 (72.8)	55 (26.7)
The number of servings recommended of dairy foods per day to meet pregnant women’s needs for calcium	50 (24.3)	155 (75.2)
The recommended iodine requirements per day for a pregnant woman	109 (52.9)	93 (45.1)
**Multiple-answer questions related to knowledge**	n (%)	
Foods that should be avoided in pregnancy to protect against listeria		
Respondents who did not know or did not tick any correct answer	28 (13.6%)	
Respondents who marked only 1 correct answer	99 (48.1%)	
Respondents who marked 2 correct answers	49 (23.8%)	
Respondents who marked all the correct answers	30 (14.6%)	
**Foods that are a good source of iron**		
Respondents who did not know or did not tick any correct answer	2 (1.0%)	
Respondents who ticked only 1 correct answer	63 (30.6%)	
Respondents who ticked 2 correct answers	82 (39.8%)	
Respondents who ticked 3 correct answers	37 (18.0%)	
Respondents who ticked all the correct answers	19 (9.2%)	
**Tips that could help reduce the effect of nausea and vomiting during pregnancy**		
Respondents who did not know or did not tick any correct answer	11 (5.3%)	
Respondents who marked only 1 correct answer	47 (22.8%)	
Respondents who marked 2 correct answers	72 (35.0%)	
Respondents who marked all the correct answers	74 (35.9%)	
**Foods that help resolve constipation during pregnancy**		
Respondents who did not know or did not mark any correct answer	-	
Respondents who marked only 1 correct answer	35 (17.0%)	
Respondents who marked all the correct answers	169 (82.0%)	

**Table 3:** The attitudes of HCP towards the significance of nutrition, their role in providing nutrition education during pregnancy, and their confidence in providing general and specific nutrition-related advice, n (%).

The Question	Very Important	Moderately Important	Important	Slightly Important	Not Important at All
How important do you think nutrition is during pregnancy?	170 (82.5)	9 (4.4)	27 (13.1)	-	-
	**Very significant role**	**Moderately significant**	**Significant**	**Slightly significant**	**Not at all significant**
How would you consider the role that HCPs can have in providing nutrition info for pregnant women?	127 (51.7)	35 (17.0)	41 (19.9)	2 (1.0)	-
	**Very confident**	**Moderately confident**	**Confident**	**Slightly confident**	**Not confident at all**
How confident would you be in talking about the following with pregnant women:					
1. General related nutrition advice	118 (57.3)	54 (26.2)	33 (16.0)	-	1 (0.5)
2. Weight gain and obesity	123 (59.7)	48 (23.3)	25 (12.1)	8 (3.9)	1 (0.5)
3. Providing advice on vitamins	129 (62.6)	43 (20.9)	24 (11.7)	1 (0.5)	2 (1.0)
4. Vegetarian diets	31 (15.0)	75 (36.4)	36 (17.5)	37 (18.0)	22 (10.7)
5. Diabetes	111 (53.9)	41 (19.9)	33 (16.0)	13 (6.3)	5 (2.4)
6. Diets of women with medical conditions	67 (32.5)	60 (29.1)	40 (19.4)	23 (11.2)	11 (5.3)
7. Post-natal nutrition (breastfeeding)	89 (43.2)	61 (29.6)	37 (18.0)	12 (5.8)	4 (1.9)
Do you prefer your patients to be:	**Underweight**	**Normal weight**	**Overweight**	**Obese**	**I do not mind any body weight**
	1 (0.5)	185 (89.6)	1 (0.5)	-	18 (8.7)
If your answer was normal weight, why would you prefer your patients to be of normal weight?	**Complications during pregnancy**	**Being bad for your reputation**	**Those women not adhering to your advice**		
	150 (72.8)	16 (7.8)	19 (9.2)		

**Table 4:** Practices of HCPs towards providing nutrition advice, talking about nutrition problems with pregnant women, and their source of info for their advice.

	n	%
**Do you provide any nutrition-related advice to pregnant women?**		
Yes	183	88.8
No	21	10.2
**On what occasions do you talk about nutrition problems with pregnant women?**		
At the first antenatal visit	27	13.1
At every antenatal visit	70	34.0
Only when the pregnant woman asks questions	16	7.8
If the pregnant woman has a medical condition requiring nutrition intervention, such as gestational diabetes	91	44.2
I only rarely discuss nutrition issues with pregnant women	2	1.0
**What is the base of information for this advice?**		
MD/Midwifery education and general knowledge	62	30.1
MD/Midwifery journals, governmental or official websites, and textbooks	37	18.0
Internet, media, and magazines	25	12.1
Other health professionals, such as dietitians	14	6.8
General knowledge, education, journals, websites, textbooks, media, magazines, and internet	66	32.0
**Do you make referrals to the dietitian?**		
Yes	149	72.3
No	25	12.1

**Table 5:** Bivariate analysis for the total score per the study variables.

	Total Score			
	Mean	Standard Deviation	*p*-Value	F
**Gender**			0.577	0.312
Female	11.88	3.08		
Male	11.63	3.17		
**Age**			0.000	7.457
21–30 years	8.33	3.24		
31–40 years	12.37	2.97		
41–50 years	12.75	3.64		
>50 years	11.20	2.60		
**Profession**			0.215	1.504
MD	11.89	3.11		
Midwife	10.00	2.83		
Hospital-based training Midwifery	10.50	4.95		
Initial midwifery degree	11.40	3.78		
**Years of experience**			0.225	1.464
<2 years	11.00	3.56		
2–5 years	10.95	3.09		
6–10 years	12.56	3.34		
>10 years	11.67	3.03		
**Territory of work**			0.093	1.920
North	12.86	2.67		
Mount Lebanon	12.16	2.70		
Beirut	11.94	3.17		
Bekaa	11.60	3.46		
South	10.21	3.51		
Nabatieh	11.57	3.10		
**Dietitian’s service**			0.179	1.817
Yes	11.90	3.33		
No	11.24	2.84		

**Table 6:** Linear regression results with total knowledge scores as dependent variable.

	Knowledge (Model 1)			Provide Nutrition Advice (Model 2)			Discuss Nutrition Issues (Model 3)		
	Standardized β Coefficient	*p*	Confidence Interval	Standardized β Coefficient	*p*	Confidence Interval	Standardized β Coefficient	*p*	Confidence Interval
Age	−0.183	0.155	(−1.420;0.227)	−0.061	0.635	(−0.098; 0.060)	0.272	0.100	(−0.026; 0.296)
Gender	−0.070	0.381	(−1.427;0.548)	−0.105	0.187	(−0.159; 0.031)	−0.065	0.552	(−0.257; 0.138)
Profession	−0.168	0.036	(−3.240;−0.114)	−0.052	0.517	(−0.201; −0.101)	−0.127	0.254	(−0.460; 0.123)
Years of experience	0.149	0.217	(−0.361;1,581)	0.128	0.286	(−0.043; 0.145)	−0.147	0.333	(−0.310; 0.106)
Dietitian’s service	−0.063	0.404	(−1.433;0.580)	−0.032	0.671	(−0.117; 0.076)	−0.029	0.786	(−0.232; 0.176)
Total score				0.216	0.003	(0.007; 0.035)	−0.270	0.009	(−0.062; −0.009)

## Data Availability

The data presented in this study are available on request from the corresponding author.
